# Selection and Expression Stability Analysis of Housekeeping Genes for Real-Time PCR Normalization in Rhesus Macaque Peripheral Blood

**DOI:** 10.3390/ani16131950

**Published:** 2026-06-24

**Authors:** Ivan R. Kumakov, Marina V. Shulskaya, Olga A. Shamsutdinova, Dmitry V. Bulgin, Maria I. Shadrina, Alexandr P. Trashkov, Petr A. Slominsky, Anelya Kh. Alieva

**Affiliations:** 1 National Research Center “Kurchatov Institute”, 1 Kurchatov sq., Moscow 123182, Russia; shulskaya.m@yandex.ru (M.V.S.); maria.i.shadrina@yandex.ru (M.I.S.); trashkov_ap@nrcki.ru (A.P.T.); paslominsky@bk.ru (P.A.S.); alieva-ah.img@yandex.ru (A.K.A.); 2 Kurchatov Complex of Medical Primatology, National Research Center “Kurchatov Institute”, Akademika Lapina Str., Veseloe Village, Sochi 354376, Russia; shamsutdinova-o-a@yandex.ru (O.A.S.); bulgin@primatologia.ru (D.V.B.)

**Keywords:** *Macaca mulatta*, transcriptome analysis, gene expression, reference genes

## Abstract

(1) Background: Although the rhesus macaque (*Macaca mulatta*) is widely maintained in captive populations for translational research, reliable molecular tools for monitoring their physiological status remain limited—a gap that directly affects population management and health monitoring. In particular, no validated panel of housekeeping genes (HKGs) is currently available for expression analysis at the mRNA level in peripheral blood of the species. (2) Methods: Peripheral blood samples from healthy male rhesus macaques aged 4–7 years were used. The expression stability of candidate HKGs was evaluated using RefFinder based on cycle threshold (Ct) values obtained from real-time PCR. (3) Results: A panel of 29 candidate HKGs was compiled. After multistage bioinformatic and experimental selection, 22 genes were excluded. Expression stability analysis of the remaining seven genes (*AHSA1*, *B4GALT3*, *CYB5R1*, *HPCAL1*, *PSMD6*, *SSR2*, and *VPS72*) identified *B4GALT3* and *PSMD6* as the most stable. (4) Conclusions: This study presents the experimentally validated set of HKGs for expression studies in *Macaca mulatta*. Based on the results of expression stability analysis, the *B4GALT3* and *PSMD6* genes can be recommended as reference genes for studying changes in gene expression in peripheral blood.

## Simple Summary

Rhesus macaques are an important model species for biological research and are also widely kept in zoos, conservation programs, and research centers worldwide. Blood tests are routinely used to monitor their health and to study how their bodies work at the molecular level. One powerful method is to measure the activity of genes from blood samples. However, to do this accurately, scientists need stable reference genes that act as a built-in ruler. Without such a ruler, the results can be misleading. For rhesus macaques, no validated set of reference genes has been available for blood studies. In this work, we collected blood from healthy male rhesus macaques, selected candidate reference genes and tested them. Using a common laboratory technique called real-time PCR, we identified two genes, *B4GALT3* and *PSMD6*, as the best suited as internal reference standards. These findings provide veterinarians and researchers with a reliable tool to accurately measure gene activity in macaque blood.

## 1. Introduction

At the present time, it has been convincingly demonstrated that changes occurring at the transcriptome level can play a significant role in the development of pathological conditions in the organism. Transcriptomic analysis plays a crucial role in investigating the fundamental basis of pathological conditions at the molecular level. In this type of research, the most common method for studying gene expression at the mRNA level is real-time PCR (qPCR). It has many advantages, such as high sensitivity, a low detection limit, a broad dynamic range spanning up to seven to eight orders of magnitude, and high specificity conferred by gene-specific primers and fluorescent probes. The method provides rapid turnaround from sample preparation to result, straightforward data analysis based on the ΔΔCt method, and low per-sample cost when only a limited number of genes are tested. However, the data obtained are relative, and stably expressed reference genes or housekeeping genes (HKGs) are required for subsequent data evaluation and processing.

Currently, a vast number of model objects exist that allow one to reproduce and simulate various human processes and conditions for further study. Although research is usually conducted using rodents, some processes and conditions are more appropriately studied in larger mammals, such as primates. For example, the rhesus macaque (*Macaca mulatta*, *M. mulatta*), a nonhuman primate, is one of the most well-characterized organisms and has phylogenetic similarities with humans compared to other large model organisms. Studying gene expression changes at the mRNA level in rhesus macaques is important both for assessing the welfare of captive macaques [1,2] and for conducting experiments using them as model animals in biological research.

Studies on human and rhesus macaque blood and tissue samples have shown that expression of the most common HKGs can vary depending on the tissue or pathological process [3,4,5,6]. In this regard, when selecting reference genes, it is necessary to check the compliance of candidate HKGs with the following criteria: absence of pseudogenes and association with the disease or condition under study and stable expression in the specific tissue under study [7]. It is important to note that genes used as HKGs in one model object may not be stably expressed in another model object [8]. In this regard, experimental testing of candidate HKGs on a new organism for research is a necessary step.

Peripheral blood is the most accessible and convenient source for transcriptome biomarker discovery, allowing for longitudinal studies, as well as testing new therapies. The available literature on HKG selection in *M. mulatta* has been analyzed in a recent bioinformatic study by Shulskaya et al. [7]. The results of the above-mentioned article are candidate HKG panels: an extended panel of 56 genes and a smaller panel of 7 genes. However, to date, no experimentally validated HKG panel exists for expression analysis in rhesus macaque peripheral blood. The present study was designed to address this gap by providing the first experimental assessment of HKG expression stability in *M. mulatta* peripheral blood.

## 2. Materials and Methods

### 2.1. Human Care Guideline

The study analyzed peripheral blood samples collected from twenty male rhesus macaques (*Macaca mulatta*) of Indian origin, 3rd–4th generation, aged 4–7 years. Power calculations were not performed, as HKG stability studies aim to rank candidate genes by expression consistency rather than to detect a predefined between-group effect size. A sample size in this range is in line with published reference gene validation studies in non-human primates [9], human peripheral blood [10], and it is sufficient to evaluate inter-individual variability in gene expression. Only male animals were included in the study. In *M. mulatta*, Urbanski et al. explicitly noted the importance of controlling for hormonal milieu in gene expression studies, particularly when female animals are involved [11]. Including females without controlling for cycle phase would therefore introduce an additional and difficult-to-standardize source of variability that could compromise the stability assessment of candidate HKGs. The potential influence of sex on HKG expression in rhesus macaque peripheral blood is noted in Limitations. All macaques resided at the Kurchatov Complex of Medical Primatology of the National Research Center “Kurchatov Institute” in Sochi, Russia. The animals were housed outdoors in group enclosures, which also had heated rooms for sleeping and shelter. Peripheral blood samples were collected from the inguinal vein using a syringe in the morning on an empty stomach. The animals fasted for 18 h before sampling, while maintaining free access to water. The blood was then immediately transferred to EDTA-containing vacutainers. Blood samples from each animal were then aliquoted (500 μL) for long-term storage at −70 °C. The experiment followed the ARRIVE 2.0 protocol [12] and was carried out in accordance with the provisions of the European Convention for the Protection of Vertebrate Animals (ETS N 123) and bioethical standards [13]. The research was approved by the Local Ethics Committee for Biomedical Research of the National Research Center “Kurchatov Institute” (protocol No. 2-2pr dated 30 June 2025).

### 2.2. Selection of TaqMan Primers and Probes

Nucleotide sequences of target genes were obtained from publicly available NCBI Gene [14] and Ensembl [15] databases. Primers were selected exclusively for conserved exon–exon spikes present in all gene isoforms using Beacon Designer 7.0 software (Premier Biosoft International, San Francisco, CA, USA). The primer selection parameters are described in the article by Partevian et al. [16]. The specificity of primers and probes was tested using the Primer-BLAST resource (Primer3 (version 2.5.0)) [17]. Primers and probes were synthesized by Evrogen (Moscow, Russia) and DNA Synthesis (Moscow, Russia), respectively. The sequences of gene-specific primers and probes are shown in Table 1.

### 2.3. Total RNA Extraction

Total RNA was extracted from 500 μL of each *M. mulatta* peripheral blood sample using a ZR Whole-Blood Total RNA Kit™ (Zymo Research Corp., Irvine, CA, USA) according to the manufacturer’s recommendations. The concentration of isolated RNA was measured using a Qubit^®^ RNA Assay Kit and a Qubit 3.0 fluorometer (Thermo Fisher Scientific, Waltham, MA, USA) according to the manufacturer’s protocol. The measurement was performed for 1 μL of the resulting total RNA sample. The concentration of all isolated total RNA was normalized to the sample with the lowest concentration (23.6 ng/μL). Samples with higher RNA concentrations were diluted to the lowest concentration and aliquoted. Yeast tRNA was added to all RNA samples after extraction [18] at a rate of 1 μL of tRNA (100 ng/μL) per 10 μL of sample. One aliquot was immediately used for the reverse transcription reaction, and the remaining aliquots were stored at −70 °C.

### 2.4. Reverse Transcription

Reverse transcription was performed using 10 μL (236 ng) of isolated total RNA, 0.6 μL of Random Hexamer Primer (Thermo Fisher Scientific, USA) and 0.4 μL of Oligo(dT)18 (Thermo Fisher Scientific, USA) as primers, and a RevertAid H Minus First Strand cDNA Synthesis Kit (Thermo Fisher Scientific, USA). The reaction was performed using a T3 Thermocycler (Biometra, Göttingen, Germany), and the following temperature cycle was used: 5 min at 25 °C, 1 h at 42 °C, 10 min at 70 °C, and a pause at 4 °C until cDNA samples were extracted. cDNA collections were stored at −20 °C.

### 2.5. qPCR (TaqMan)

A QuantStudio 3 instrument (Thermo Fisher Scientific, USA) was used for qPCR. cDNA obtained by reverse transcription was used as a template. The 30 μL reaction mixture contained: 12.8 μL of MQ water, 5 μL of cDNA template (0.02 ng/μL) diluted 50-fold with an aqueous solution of yeast tRNA [18], 3 μL PCR buffer (10×) (Synthol, Moscow, Russia), 3 μL 25 mM MgCl2, 3 μL primer mixture (10 pmol/μL) and probes (2.5 pmol/μL), 3 μL dNTPs (2 nmol/μL each) (Thermo Fisher Scientific, USA), and 0.2 μL SynTaq DNA polymerase (5 units/μL) (Synthol, Russia). The following temperature cycle was used for the samples during qPCR: 60 s at 50 °C, 40 cycles (95 °C 15 s, 61 °C 30 s), 30 s at 25 °C. Each qPCR reaction was carried out in three parallel technical replicates.

### 2.6. Statistical Data Processing

The stability of HKGs’ expression was assessed using the Ct amplification threshold cycle values, MS Excel 2021 (Microsoft Corporation, Redmond, WA, USA), and the RefFinder web tool [19]. The latest tool integrates results from four independent algorithms to determine the stability of expression of HKGs (geNorm [20], NormFinder [21], BestKeeper [22], and ΔCt [23]) by calculating the geometric mean of their ranks. This approach allows us to obtain a single ranked list of reference genes that minimizes the systematic errors inherent in each of the individual algorithms.

## 3. Results

A multistage analysis was conducted to select the optimal HKGs for the expression studies at the mRNA level in *M. mulatta* peripheral blood. Earlier, we compiled a panel of the 25 most promising HKGs for *M. mulatta* peripheral blood studies [7]. The orthologs of HKGs, stably expressed in both humans and mice (*AARS*, *POLR2F*, *PSMD6*, and *SARS1*) were also included in the list of candidates [8]. Thus, the initial panel included 29 genes (*AARS*, *AHSA1*, *ARHGDIA*, *ARL2*, *BAGALT3*, *CSNK2B*, *CYB5R1*, *DIAPH1*, *GPX4*, *H6PD*, *HPCAL1*, *LDHA*, *MAPKAPK2*, *NDUFA1*, *NDUFA7*, *POLR2F*, *PRPF8*, *PSMD6*, *RAD23A*, *RRAGA*, *SARS1*, *SSR2*, *TBP*, *TMED9*, *TTC1*, *UBA6*, *UBB*, *VPS72*, and *YWHAH*).

Since we selected HKGs for the study of age-related changes, nine genes (*CSNK2B*, *DIAPH1*, *GPX4*, *LDHA*, *MAPKAPK2*, *NDUFA1*, *RAD23A*, *TBP*, and *UBB*) that may be associated with age-dependent changes were excluded from this list at the first stage [7].

The next selection step excluded genes without confirmed sequences in the NCBI Gene database for *M. mulatta* (*AARS*, *ARHGDIA*, *ARL2*, *H6PD*, *NDUFA7*, *PRPF8*, *SARS1*, and *UBA6*). Additionally, the *RRAGA* and *YWHAH* genes were excluded, as primer systems for them could not be selected to meet the specified criteria; these are described in detail in the article “Screening and stability analysis of housekeeping genes for transcriptomic studies for *Danio rerio* (Zebrafish) at early developmental stages” [16]. Thus, the final list of HKGs for experimental verification of expression stability included ten genes (*AHSA1*, *B4GALT3*, *CYB5R1*, *HPCAL1*, *POLR2F*, *PSMD6*, *SSR2*, *TMED9*, *TTC1*, and *VPS72*).

TaqMan primer and probe systems were selected for the remaining ten genes. Confirmation of specific amplicons during the PCR reaction and the absence of nonspecific reaction products was performed using agarose gel electrophoresis.

When testing the primer systems selected for the *POLR2F*, *TMED9*, and *TTC1* gene sequences, no specific product was detected. Therefore, these HKGs were excluded from further work. Thus, after selecting the TaqMan systems, we obtained a final list of seven genes (*AHSA1*, *B4GALT3*, *CYB5R1*, *HPCAL1*, *PSMD6*, *SSR2*, and *VPS72*), for which qPCR with TaqMan probes was performed.

For these genes, Ct values were obtained during qPCR and used to assess expression stability using the RefFinder web tool.

The HKG that has the lowest value for the “Final score” indicator is the most stably expressed. The combined results of the HKGs expression stability analysis and their final ranking are presented in Table 2.

As can be seen from the presented data, the most stable expression in the peripheral blood of *M. mulatta* is observed for *B4GALT3* and *PSMD6* genes.

## 4. Discussion

The rhesus macaque is a promising model for various biomedical studies, including those at the transcriptome level. The use of stably expressed HKGs is necessary to obtain valid results when analyzing gene expression at the mRNA level. However, experimentally validated data on such HKGs in the peripheral blood of *M. mulatta* are currently lacking. We compiled a list of 29 promising HKGs for transcriptome studies. This list included 25 genes taken from the article by Shulskaya et al. [7] and four HKGs orthologs with confirmed stable expression in humans and mice [8].

In the first stage, candidate HKGs were screened for associations with neurodegenerative diseases on the basis of further research prospects. Thus, nine candidate HKGs that could be associated with neurodegeneration were excluded from further study [7]. It is worth noting that the list of genes excluded at this stage depends on the pathology being studied and is subject to change.

Furthermore, eight more genes from this list were excluded from further work because they did not have a confirmed sequence and had a PREDICTED status in the NCBI Gene database. This status indicates that the gene was identified using computer analysis and has not yet been confirmed experimentally. At the same time, a confirmed sequence is important to eliminate the risk of false positives and increase the reliability of the experimental data. Therefore, one of the selection criteria was the presence of a PROVISIONAL, VALIDATED, or REVIEWED status.

During the TaqMan system selection process, two genes were excluded based on their exon–intron structure: *RRAGA*, which has a single exon, and *YWHAH*, for which it was impossible to select a primer system positioned on opposite sides of the exon junction. When selecting a TaqMan system for qPCR, it is crucial to ensure that primers are positioned on opposite sides of the exon–exon junction. This primer arrangement ensures efficient amplification of cDNA only, as it lacks the introns that separate exons in genomic DNA, thereby allowing cDNA to be distinguished from genomic DNA. This approach eliminates the need for DNase treatment of RNA samples, which can potentially affect the quality and quantity of RNA, which is critical when working with unique samples, including peripheral blood with relatively low RNA concentrations. Despite having PROVISIONAL status and the ability to select a primer system that met all criteria, the *POLR2F*, *TTC1*, and *TMED9* genes were excluded from further analysis. This exclusion was caused by the lack of an amplification product of the expected length when using the selected primers. It may possibly be explained by the fact that the genome of *M. mulatta* remains insufficiently annotated. Inaccurate primary structure sequences, errors in exon-intron structure annotation, and incorrect coding region identification may lead to primer dimerization and accumulation of a nonspecific reaction product.

As can be seen from Table 2, different algorithms may rank genes differently. According to the ΔCt and NormFinder algorithms, the most stably expressed genes are *HPCAL1* and *B4GALT3*. However, according to BestKeeper, *B4GALT3* and *PSMD6* are considered the most stably expressed, while *HPCAL1* ranks fifth. When analyzing the results of all four algorithms together, the most stably expressed genes are *B4GALT3* and *PSMD6*. This difference in ranking by the above-mentioned algorithms indicates that the generalization of the results of these algorithms, as performed by RefFinder, facilitates a more accurate assessment of the stability of HKGs expression, which is independent of the specific algorithm.

The processes and functions associated with proteins encoded by candidate HKGs are presented in Table 3.

According to the results of our analysis, the most stable expression is found for the *B4GALT3* gene, which encodes an enzyme involved in the synthesis of complex N-linked oligosaccharides in many glycoproteins, as well as carbohydrate components of glycolipids [24]. There is experimental evidence indicating changes in *B4GALT3* expression in cancer [25,26]. This makes it unsuitable as an HKG for transcriptomic studies related to this pathology. However, this gene can be used as a reference gene for other non-oncology-related studies.

Another stably expressed gene is *PSMD6*, which encodes a component of the 26S proteasome, a multiprotein complex involved in the ATP-dependent degradation of ubiquitinated proteins. An ortholog of this gene is stably expressed in human peripheral blood and mouse brains [8]. This study confirms that the *PSMD6* gene can be used as an HKG for transcriptomic studies in *M. mulatta*. It is important to note the process in which the protein encoded by this gene is involved. Proteasome degradation is a fundamental cellular process for maintaining protein homeostasis. Mutations in the genes encoding proteasome subunits or disruptions in the stability of their expression can lead to various pathological conditions [27]. *PSMD6* has not been associated with nervous system disorders and, therefore, can be recommended as an HKG for research in this area.

It is noteworthy that HKG has experimentally confirmed stable expression in the tissue of one organism; this does not mean that this gene can be used as an HKG without experimental confirmation of its stable expression in another study subject. For example, *AARS* and *SARS*, despite stable expression in humans and mice, do not have a confirmed sequence in *M. mulatta*. Therefore, these genes cannot be used as reference genes for gene expression analysis in macaques. Overall, as this study showed, only *PSMD6*, one of the four genes selected as HKG orthologs in other model organisms, passed the experimental validation stage.

The most stably expressed HKGs selected in this study were primarily chosen for the study of age-related changes. However, the use of these genes as reference genes can also be recommended for studies examining other changes or pathologies not associated with cancer.

Beyond the specific gene panel identified here, the multistage workflow applied in this study—comprising bioinformatic pre-screening for candidates and filtering by expression level and disease association, followed by experimental stability assessment across multiple algorithms—constitutes a reproducible framework that can be adapted for HKG selection in other tissues, experimental conditions, or non-human primate species. Researchers working in related contexts may find this approach useful as a starting point for context-specific reference gene validation, even if the particular genes identified in rhesus macaque peripheral blood require re-evaluation under different conditions.

### Limitations

The findings of this study are applicable within the specific conditions under which it was conducted. The analysis was performed on peripheral blood samples from healthy male *M. mulatta* of Indian origin. The inclusion of males only was motivated by the need to eliminate the confounding effect of cyclical hormonal fluctuations in females. The study included only primates aged 4–7 years. The expression stability of the identified HKGs cannot be automatically assumed to extend to other tissues, different experimental conditions, or animals of a different sex, age, health status, or geographical origin—in all such cases, prior validation using one’s own research material is strongly recommended.

## 5. Conclusions

An experimentally validated set of HKGs that can be used for gene expression studies at the mRNA level in the peripheral blood of *M. mulatta* was generated. Analysis of the expression stability of candidate HKGs revealed that the *B4GALT3* and *PSMD6* genes exhibited the greatest expression stability compared to other analyzed genes and can be recommended as reference genes for transcriptomic studies of diseases and conditions in peripheral blood. Since altered *B4GALT3* expression has previously been demonstrated in cancer, this gene cannot be used as a reference gene when modeling such pathologies in macaques.

It should be noted that *PSMD6* is a stably expressed HKG not only in the peripheral blood of *M. mulatta* but also, as previously shown, in human peripheral blood and mouse brains. Therefore, when selecting HKGs for expression analysis of other model objects, it is advisable to include this gene in the study.

## Figures and Tables

**Table 1:** Sequences of gene-specific TaqMan primers and probes.

Gene	Nucleotide Sequences		Tm, °C	Amplicon Length, bp
*AHSA1* NM_001266884.1 ^1^	Probe	5′-VIC ^2^-TGGAGCGTCAAACTAAACTGGACAGGTACT-BHQ2 ^3^-3′	67.29	122
	Forward primer	5′-AATCGCAAAGGGAAACTTATCTTCT-3′	59.05	
	Reverse primer	5′-TTTTCATCAGACAAATTGGGGATCT-3′	58.98	
*B4GALT3* NM_001257845.1	Probe	5′-VIC-CGCACCCCAACATTGAGCAGTTTTGC-BHQ2-3′	67.74	111
	Forward primer	5′-GCTTATGGCATCTATGTCATCCA-3′	58.42	
	Reverse primer	5′-CAGGCAGTCCCACTCTTCA-3′	58.94	
*CYB5R1* NM_001257476.2	Probe	5′-VIC-AGCATTGGGGTGATTCCTGTCCCGC-BHQ2-3′	69.08	171
	Forward primer	5′-TCACTTACACTGGAAAAGGGCA-3′	59.83	
	Reverse primer	5′-GAAAGCACTGGGTTGGATCTTC-3′	59.51	
*HPCAL1* NM_001260988.1	Probe	5′-VIC-GGGGTCGCTCTTGGCACCTTTGATGAAT-BHQ2-3′	69.00	131
	Forward primer	5′-CAGGCAGATGGACACCAACAA-3′	60.82	
	Reverse primer	5′-GCCGCTCGCTCAGAACTG-3’	61.18	
*POLR2F* NM_001265974.1	Probe	5’-VIC-ATGACCAAGTACGAGCGAGCCCG-BHQ2-3’	66.78	193
	Forward primer	5’-GCCAACCAGAAGCGAATCAC-3’	59.83	
	Reverse primer	5’-GCAGGTAACGGCGAATGATG-3’	59.70	
*PSMD6* NM_001266757.1	Probe	5’-VIC-CGAGTTGAAGCGTTTGGATGAGGAGC-BHQ2-3’	66.22	182
	Forward primer	5’-CGTGGACCTACTCAATAAAATGAAGA-3’	59.18	
	Reverse primer	5′-TCTTGCGAAAGGCTGTCAGA-3′	59.61	
*SSR2* NM_001257621.1	Probe	5′-VIC-CCCGTTGTGATTGGCTCTACCAGTGC-BHQ2-3′	67.66	141
	Forward primer	5′-CACCTCGGCGACAATTACTTAC-3′	59.40	
	Reverse primer	5′-GCTGCCCAGTCCAGAAAATG-3′	59.47	
*TMED9* NM_001193984.2	Probe	5′-VIC-GTGAACCTGCCCTCGGAGCCATACT-BHQ2-3′	68.09	118
	Forward primer	5′-GTGAAGGACCCAGAGGACAAG-3′	60.00	
	Reverse primer	5′-TGGTGGAATTGGAGTGAAGACA-3′	59.56	
*TTC1* NM_001266114.1	Probe	5′-VIC-GCAGCAAAGCAATTCAGTTAAACCCCAGC-BHQ2-3′	67.50	116
	Forward primer	5′-CAAGGATGAAACAGGACAAGAAAGAA-3′	59.96	
	Reverse primer	5′-CGTCTTCTCATACAACTCCGCT-3′	60.16	
*VPS72* NM_001266143.1	Probe	5′-VIC-ACGCATGGACTTCCTACTGTCAGAGCC-BHQ2-3′	67.92	135
	Forward primer	5′-GCACTACTGCCACTAGAACTACA-3′	59.81	
	Reverse primer	5′-CCCCTTTCGCCGTCTTGA-3′	59.65	

^1^ NM—mRNA sequence identifier (NCBI Reference Sequence), ^2^ VIC—fluorescent dye, ^3^ BHQ2—Black Hole Quencher—fluorescence quencher.

**Table 2:** Rating of HKGs for the expression analysis at the mRNA level in peripheral blood of *M. mulatta*.

Total Rating	HKG ^1^	Algorithm				Final Score ^2^
		ΔC_t_	BestKeeper	NormFinder	geNorm	
1	*B4GALT3*	0.75	0.57	0.40	0.44	1.19
2	*PSMD6*	0.77	0.62	0.43	0.44	2.06
3	*HPCAL1*	0.76	0.85	0.32	0.58	2.52
4	*AHSA1*	0.80	0.70	0.79	0.49	3.46
5	*CYB5R1*	0.93	0.75	0.67	0.71	5.18
6	*SSR2*	0.91	1.08	0.68	0.64	5.48
7	*VPS72*	1.35	1.09	1.25	0.90	7.00

^1^ Housekeeping gene, ^2^ The final score is a rating compiled based on the scores from all four algorithms.

**Table 3:** Functions of proteins encoded by candidate HKGs.

Total Rating	Protein Encoded by HKG	Function
1	B4GALT3	Involved in the synthesis of complex N-linked oligosaccharides in many glycoproteins, as well as carbohydrate components of glycolipids
2	PSMD6	Encodes a component of the 26S proteasome, a multiprotein complex involved in the ATP-dependent degradation of ubiquitinated proteins
3	HPCAL1	Neuron-specific Ca^2+^-binding protein involved in calcium-dependent regulation of rhodopsin phosphorylation and neuronal signaling
4	AHSA1	Activator of HSP90 ATPase activity
5	CYB5R1	NADH-cytochrome b5 reductase 1 which participates in desaturation and elongation of fatty acids, cholesterol biosynthesis and drug metabolism
6	SSR2	Subunit of the β receptor of the signal sequence (SSR). Provides cotranslational translocation of proteins into EPR
7	VPS72	Participation in the work of chromatin-remodeling complexes; Histone chaperone specific to H2A.Z

## Data Availability

Data is contained within the article.

## Abbreviations

The following abbreviations are used in this manuscript:

qPCR	Real-time PCR
HKG	Housekeeping gene
